# Decreasing Pasteurization Treatment Efficiency against Amoeba-Grown *Legionella pneumophila*—Recognized Public Health Risk Factor

**DOI:** 10.3390/ijerph19031099

**Published:** 2022-01-19

**Authors:** Maša Knežević, Dobrica Rončević, Darija Vukić Lušić, Mirna Mihelčić, Rok Kogoj, Darja Keše, Marin Glad, Arijana Cenov, Mateja Ožanič, Daniela Glažar Ivče, Marina Šantić

**Affiliations:** 1 Department of Microbiology and Parasitology, Faculty of Medicine, University of Rijeka, Braće Branchetta 20, 51000 Rijeka, Croatia; masa.knezevic@uniri.hr (M.K.); mirna.mihelcic@uniri.hr (M.M.); mateja.ozanic@uniri.hr (M.O.); marina.santic@uniri.hr (M.Š.); 2 Department of Epidemiology, Teaching Institute of Public Health of Primorje-Gorski Kotar County, Krešimirova 52a, 51000 Rijeka, Croatia; dobrica.roncevic@zzjzpgz.hr; 3 Department of Public Health, Faculty of Health Studies, Viktora Cara Emina 5, 51000 Rijeka, Croatia; 4 Department of Environmental Health, Faculty of Medicine, University of Rijeka, Braće Branchetta 20, 51000 Rijeka, Croatia; 5 Department of Environmental Health, Teaching Institute of Public Health of Primorje-Gorski Kotar County, Krešimirova 52a, 51000 Rijeka, Croatia; marin.glad@zzjzpgz.hr (M.G.); arijana.cenov@zzjzpgz.hr (A.C.); 6 Center for Advanced Computing and Modeling, University of Rijeka, Radmile Matejčić 2, 51000 Rijeka, Croatia; 7 Institute of Microbiology and Immunology, Faculty of Medicine, University of Ljubljana, Zaloska 4, 1000 Ljubljana, Slovenia; Rok.Kogoj@mf.uni-lj.si (R.K.); Darja.Kese@mf.uni-lj.si (D.K.); 8 Branch Office Rab, Teaching Institute of Public Health of Primorje-Gorski Kotar County, Palit 143a, 51280 Rab, Croatia; daniela.glazar-ivce@zzjzpgz.hr

**Keywords:** *Acanthamoeba*, environment, infection, *Legionella*, water

## Abstract

*Legionellae* are gram-negative bacteria most commonly found in freshwater ecosystems and purpose-built water systems. In humans, the bacterium causes Legionnaires’ disease (LD) or a Pontiac fever. In this study, the different waters (drinking water, pool water, cooling towers) in which *Legionella pneumophila* has been isolated were studied to assess the possible risk of bacterial spreading in the population. The influence of physical and chemical parameters, and interactions with *Acanthamoeba castellanii* on *L. pneumophila*, were analyzed by Heterotrophic Plate Count, the Colony-forming units (CFU) methods, transmission electron microscopy (TEM), and Sequence-Based Typing (SBT) analysis. During the study period (2013–2019), a total of 1932 water samples were analyzed, with the average annual rate of *Legionella*-positive water samples of 8.9%, showing an increasing trend. The largest proportion of *Legionella*-positive samples was found in cooling towers and rehabilitation centers (33.9% and 33.3%, respectively). Among the isolates, *L. pneumophila* SGs 2–14 was the most commonly identified strain (76%). The survival of *Legionella* was enhanced in the samples with higher pH values, while higher electrical conductivity, nitrate, and free residual chlorine concentration significantly reduced the survival of *Legionella.* Our results show that growth in amoeba does not affect the allelic profile, phenotype, and morphology of the bacterium but environmental *L. pneumophila* becomes more resistant to pasteurization treatment.

## 1. Introduction

*Legionellae* are gram-negative bacteria commonly found in freshwater ecosystems such as rivers, lakes, ponds, and streams [1,2]. The entry of bacteria into purpose-built water systems and drinking-water distribution systems make a serious health concern. After the growth and replication of bacteria in drinking-water distribution systems, people can be infected by aspiration of water droplets. In humans, the bacteria replicate in alveolar macrophages, causing a serious type of pneumonia known as Legionnaires’ disease (LD) or a mild disease called Pontiac fever [3,4]. Around 90% of Legionnaires’ disease cases are caused by the *L. pneumophila* serogroup 1. *L. pneumophila* serogroups 2–14 have been isolated from more than 50% of man-made water systems and cause only 15 to 20% of community-acquired pneumonia [5].

Natural water bodies constitute the largest reservoir of *Legionella*, but the bacteria are also found in many types of water equipment such as plumbing systems, cooling towers, water systems in hotels, households, water tanks, decorative fountains, whirlpool spas, hospital equipment, and showerheads [6,7,8]. In these systems, water stagnation, warm temperatures, chemical constituents, and aged plumbing can affect the proliferation of bacteria. Water temperature is a very important factor in the regulation of the microbial growth curve, disinfection efficiency, and corrosion rates [9]. *L. pneumophila* multiplies in a temperature range of 20–48 °C, but the optimal temperature for bacterial growth is 35 °C [10]. Moreover, various physical and chemical factors can affect the survival and growth of bacteria, such as pH, nutrients, disinfection concentrations, and the presence of heavy metals in the water [11,12].

Bacteria are often in close association with freshwater protozoa [13,14]. The interactions between *L. pneumophila* and free-living amoebae are very important for the pathogenesis of the bacterium [15]. Amoeba-grown *L. pneumophila* has shown various phenotypic modulations, including enhanced entry and replication in monocytes and increased virulence in mice, compared to bacteria grown on agar [16]. Under various adverse or stress conditions, free-living amoebae differentiate from the vegetative form called trophozoites to the resting form called cyst [17]. It is assumed that the survival of *L. pneumophila* in *Acanthamoeba* cysts may be a mechanism used by the bacterium to avoid treatment with disinfectants and then spread to nature [18]. *Legionella* can also be found inhabiting mixed-community biofilms, which affects the survival of bacteria and protects them from harmful compounds [19].

The disinfecting strategies of water distribution systems include thermal shock, UV light, copper-silver ionization, hyper chlorination, chlorine dioxide, monochloramine, and point-of-use filters [20]. Despite regular disinfection of water systems, cases of legionellosis continue to appear and they could be related to long-term survival of bacteria in amoebae.

Numerous studies have shown that the spread of bacteria in water distribution systems may be responsible for community-acquired cases of LD. It was previously demonstrated that *Legionella* infections in sports centers can be transmitted after the use of hot water systems such as showers or swimming pools [21]. A study covering 31 EU/EEA countries showed that the sites where *Legionella* infection occurred had frequent recurrent infections within the following 2 years even though effective control measures had been implemented [22]. In a study conducted from 2011 to 2015 in 30 European countries, 30,532 cases of Legionnaires’ disease were reported with an annual average increase of 0.09 cases/100,000 people (95% CI 0.02–0.14; *p* = 0.02), of which 70.3% were in France, Germany, Italy, and Spain, although their population accounted for only 49.9% of the total population [5]. In the reported cases, 70.7% of the infections were community-acquired, 19.9% were travel-associated, and 7.3% were healthcare-related diseases. In an outbreak of LD in Portugal in 2014, out of 403 identified cases, 71 clinical isolates had an identical ST1905 profile as the industrial cooling tower system isolate [23]. A previous 3-year monitoring study of *L. pneumophila* carried out in Southern Croatia revealed the presence of *L. pneumophila* in 32.6% of the investigated water samples, mainly from taps in hotels and homes for the elderly and disabled. The water samples that tested positive for *L. pneumophila* exhibited higher Cu, Fe, Mg, and Ca concentrations, and lower concentrations of Mn compared to the negative samples [24].

The primary focus of this study was to investigate the frequency of colonization by *L. pneumophila* in different water sources (pools, wellness centers, hotels, apartments, camping sites, rehabilitation centers, ferries, and cooling towers) in the Primorje-Gorski Kotar County, Croatia. A second aim of the study was to identify SBT types and phenotypes of environmental isolates and investigate whether a change would occur in the allelic profile, phenotype, or resistance of bacteria after cultivation in amoeba. The influence of physical–chemical parameters and interactions of *L. pneumophila* with amoebae were analyzed to identify possible factors that can contribute to reduction in human infection.

## 2. Materials and Methods

### 2.1. Samples and Amoeba Culture

A total of 1932 water samples were taken from swimming pools (public and spa), drinking-water distribution systems (hotels, apartments, camping sites, rehabilitation centers, ferries), and cooling towers in Primorje-Gorski Kotar County. Public water supply in Primorje-Gorski Kotar County is based on groundwater and surface water resources. Groundwater was used in cooling towers and swimming pools are filled with water from the groundwater system. All genotyped *Legionellae* came from groundwater or surface water samples (see paragraph 3.4. for details). The samples were collected as pre-flush samples, without flushing and without flaming the outlet (in order to define the bacterial colonization at a specific outlet point). The samples were placed in 1-litre sterile bottles, with 10% sodium thiosulphate (1 mL/L) in order to neutralize the chlorine or other halogen-based biocides. The samples were protected and transported to the laboratory in appropriate cooled containers, at a temperature of 2–8 °C, and tested for the presence of *Legionella pneumophila* within 24 h. After detection in environmental water samples, *Legionella pneumophila* was grown on Buffered Charcoal Yeast Extract agar (BCYE). *Legionella pneumophila* AA100 was used as a positive control.

*Acanthamoeba castellanii* was obtained from the American Type Culture Collection, 30234, and cultivated in peptone–yeast extract–glucose medium (PYG = ATCC medium 712) at 25 °C.

### 2.2. Microbiological Analyses

#### 2.2.1. Detection and Quantification of *Legionella* spp. in Water Samples

The isolation of *Legionellae* and the estimation of their number in different types of water samples was carried out using the culture method, according to ISO 11731:2017 [25]. Over the period from 2013 to 2016, *L. pneumophila* was analyzed in agreement with ISO 11731:1998, which is the previous version of the current standard, albeit, the procedure remained unmodified. Filtration was used to concentrate *Legionella* in water samples. One liter of water was filtered through a polycarbonate membrane filter (0.2 μm pore-size, diameter 47 mm, Pall Corporation, Port Washington, NY, USA). Then, the membrane filter was transferred to a sterile container filled with 10 mL of distilled water. In order to enhance elution, the membrane was fragmented into smaller pieces using sterile scissors. It was placed in an ultrasonic water bath at 35 Hz/2 min (to detach the bacteria from the surface of the membrane filter).

Aliquots of 0.1 mL of the water samples subjected to heat treatment were spread onto culture-selective media, namely, Glycine Vancomycin Polymyxin B Cycloheximide (GVPC) agar (OXOID, UK). Inoculated plates were inverted and incubated in humidified air with 2.5% CO_2_ at 36 ± 1 °C for 7–10 days. The plates were inspected every 2–3 days. Presumptive colonies were taken and sub-cultured on plates of the following media: (1) BCYE, and (2) Buffered Charcoal Yeast Extract without L-cysteine (BCYE-Cys) (OXOID, UK), or other suitable culture media (for example, sheep blood agar—sheep BA). The media were incubated at 36 ± 1 °C for more than 2 days. Growth of morphologically characteristic bacterial colonies on the BCYE medium, and no growth on the BCYE-Cys, were presumed to be *Legionella*. This commercial test enables identification of different serogroups of *L. pneumophila*, serogroup 1, serogroups 2–14, as well as seven additional *Legionella* species considered important for human infection. The limit of detection of the *Legionella* quantification method was 100 CFU/L.

#### 2.2.2. Determination of Heterotrophic Plate Count (HPC)

Heterotrophic Plate Count (HPC) was counted in two ways: (1) according to ISO 6222:1999 [26], which is routinely used in water microbiology with Yeast Extract Agar (YEA, Biolife, Italy), incubated at 36 °C ± 2 °C for 44 ± 4 h and at 22 °C ± 2 °C for 68 ± 4 h, and (2) by means of sheep Blood Agar—BA (Liofilchem, Italy), incubated at 36 °C ± 2 °C during 44 ± 4 h and 96 ± 4 h.

In both cases, 1 mL (or appropriate dilution) of a water sample was taken and spread using the poured plate method. The results were calculated as the number of colonies in mL (CFU/mL). Separate counts were made for two culture medium types, each of them under two different incubation conditions.

### 2.3. Physical–Chemical Parameters

Water temperature was measured using a calibrated alcohol-filled thermometer, temperature range between 1 and 90 °C, 0.1 °C/graduation, according to the Standard Method 2550 B [27]. The concentration of residual free chlorine was determined according to ISO 7393-2:2018 [28], using a portable Colorimeter™ II (Hach, CO, USA), with a quantification limit of 0.02 mg Cl_2_/L.

The S47 instrument (SevenExcellence, Mettler Toledo, Germany) was used to record electrical conductivity, with a quantification limit of 9 µS/cm, and pH value of the water according to ISO 7888:1985 [29] and ISO 10523:2008 [30], respectively.

### 2.4. Infection of Amoeba with L. pneumophila

The number of *A. castellanii* cells (10^6^ cells per mL) was determined using a Neubauer chamber [31]. The amoeba cells were transferred in six-well plates and incubated overnight at 27 °C in 3 mL of media. On the following day, the amoeba cells were infected [32] with *L. pneumophila* strains isolated from various water samples and control bacteria, *L. pneumophila* AA100, at a multiplicity of infection (MOI) of 100, centrifuged at 240× *g* for 3 min at room temperature to synchronize the infection, and then incubated at 27 °C for 6 h. After incubation, the amoeba cells were washed three times with ATCC glucose-free media to remove extracellular bacteria. This was considered as the initial incubation time (time zero). At certain points in time, after infection (0, 2, 4, 6, 8, and 10 h), the amoeba cells were treated with 0.1% Triton X-100 (Sigma, St. Louis, MO, USA [33]) for 10 min to lyse the amoeba cells. The number of bacteria cultured in amoeba was determined by the CFU method, by adding serially diluted suspensions of *Legionella* on BCYE agar plates, and incubation for 72 h at 37 °C. The bacteria cultured in amoeba for 4 days were used in pasteurization trials.

### 2.5. Preparation of Bacterial Suspensions

For the preparation of bacterial suspensions, six representative *L. pneumophila* samples were selected (autocamp (cold water), autocamp (warm water), hotel, apartment, ferry, and cooling tower). *L. pneumophila* AA100 was used as a control. To prepare the agar-grown *Legionella* samples, *L. pneumophila* were grown on BCYE agar plates at 35 ± 2 °C for 72 h. For the preparation of the *A. castellani*-grown *Legionella* samples, *L. pneumophila* isolates were incubated in amoeba for 96 h at 27 °C, as described above, and treated with 0.1% Triton X-100 for 10 min to lyse the amoeba cells. Bacterial suspensions from amoeba-grown samples were prepared immediately after being isolated from the amoeba. The agar and amoeba-grown bacterial suspensions were prepared in sterile water, and then measured by spectrophotometry to yield a standard suspension containing approximately 10^9^ *Legionellae* per mL.

### 2.6. Pasteurization Treatments

The agar-grown or *A. castellanii*-grown *L. pneumophila* were used in a pasteurization treatment. A 5 µL measure of standard suspension was added to tap water samples (5 mL) to yield inoculated water samples containing 10^6^ CFU of *L. pneumophila* per mL for use in pasteurization trials. Water samples were pasteurized at the laboratory as follows. Aliquots (1 mL) of inoculated tap water samples containing 10^6^ CFU/mL of *L. pneumophila* were heated in thermoblock operating at different temperatures and time frames (55 °C/10 min, 53 °C/10 min, 50 °C/10 min, 50 °C/30 min). After the appropriate holding time, the tubes were placed on ice for rapid cooling. The number of bacteria after the pasteurization treatment was determined by the CFU method.

### 2.7. Transmission Electron Microscopy (TEM)

TEM analysis was performed to assess the morphological and structural changes in bacteria after the pasteurization treatment or growth in amoeba. The samples were prepared for TEM on carbon-coated Cooper grids (SPI Supplies, USA) using 2% phosphotungstic acid for staining. The *L. pneumophila* suspension (10 µL) was applied to the grid for 2 min and then removed from the grid using filter paper. Subsequently, 10 µL of acid was added to the grid for 1 min, and then drained using filter paper. The grids were air-dried before use, and 10 fields of each grid were randomly observed under a TEM device (Zeiss 902A).

### 2.8. Sequence-Based Typing (SBT) Analysis

A Wizard Genomic DNA Purification Kit (Promega, USA) was used for *L. pneumophila* DNA extraction, according to the instructions provided by the manufacturer. The EWGLI standard protocol [34,35] was used for amplification of the seven gene loci (*flaA*, *mompS*, *proA*, *asd*, *mip*, *pilE*, *neuA*).

A BigDye Terminator v3.1 Cycle Sequencing Kit (Applied Biosystems, USA) was used for Sanger sequencing in an ABI 3500 Genetic Analyzer (Applied Biosystems, Foster City, CA, USA) according to the instructions of the manufacturer. Sequencing PCR reaction clean-up was performed using a BigDye XTerminator Purification Kit (Applied Biosystems, USA), according to the manufacturer’s instructions.

Sequencing data obtained with both primers were aligned using the CLC Main Workbench version 7.9.1 (QIAGEN Aarhus S/A, Germany), and consensus sequences were produced. The consensus sequences were then entered into the EWGLI *Legionella* SBT database (Public Health England) in order to determine respective allele numbers. The combination of all seven allele numbers was queried in the database for each isolate, and returned as the Sequence-Based Type (ST) number.

### 2.9. Phenotypic Characterization

*L. pneumophila* serogroup specificity is conferred by the lipopolysaccharide structure. Using monoclonal antibodies (MAbs), some serogroups can be further subdivided into MAb subgroups of the Dresden panel by IFA test. The analysis divided *L. pneumophila* SG 1 into the following subgroups: Knoxville, Philadelphia, France/Allentown, Benidorm, OLDA, Oxford, Heysham, Camperdown, and Bellingham [36]. A positive reaction was detected by green fluorescence revealed by an Eclipse E400 (Nikon, Tokyo, Japan) fluorescence microscope.

### 2.10. Statistical Analysis

Descriptive statistics were used to present the following data: relative frequency, mean value (MV) and median, standard deviation (SD), standard error (SE), interquartile range (IQR), and data range (MIN–MAX) as measures of data dispersion; the data were also presented graphically. The normality of data distribution was tested using the Kolmogorov–Smirinov test. Logarithmic transformation of microbiological data was carried out to normalize non-normal distributions. When obtained, the data did not follow normal distribution; nonparametric tests—Spearman’s Correlation Coefficient, Mann–Whitney U test (M–W U)—were performed using the TIBCO Statistica 13.5.0 software package (TIBCO Software Inc., Palo Alto, CA, USA). Statistical significance was set at 0.05. Student’s *t*-test, * *p* < 0.05, ** *p* < 0.01, *** *p* < 0.001 were considered significantly different in comparison to the control sample.

## 3. Results

### 3.1. Legionella Prevalence

The prevalence of *Legionella* spp. in water distribution systems was examined during the 2013–2019 period in Primorje-Gorski Kotar County, Croatia [37]. The number of analyzed samples ranged from 93 in 2014 to 511 in 2019. In the first three years of the study period (2013–2015), *Legionella* spp. was not detected. From 2016 to 2019 the average percentage of *Legionella*-positive samples was 12.0%, with the largest value of 14.6% recorded in 2018 (Table 1).

**Figure 1:**
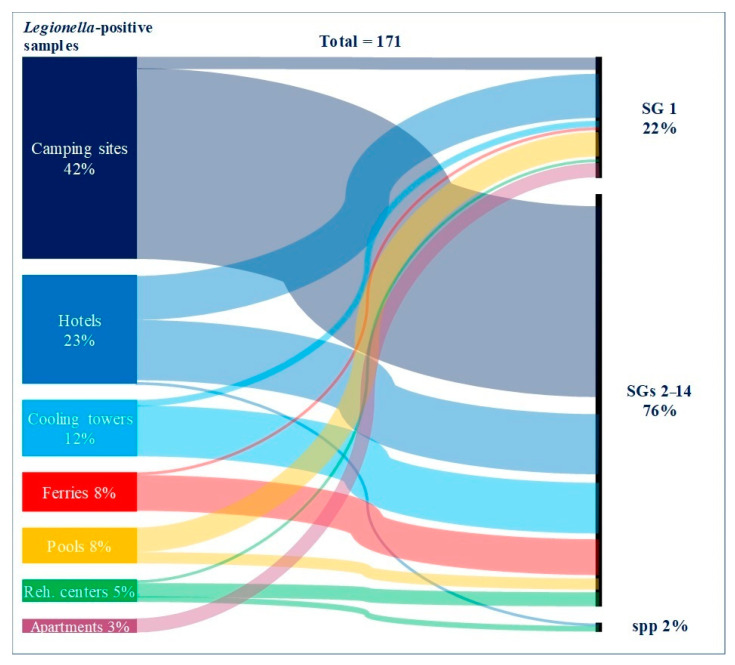
Proportion of *L. pneumophila*-positive and *Legionella* spp.-positive samples by facilities with distribution of *L. pneumophila* serogroups (SG 1 vs. SGs 2–14). The thickness of the color pattern is related to the proportion of *Legionella*-positive samples.

Of the total 1932 water samples analyzed, 171 (8.9%) were *L. pneumophila* positive. Samples were taken primarily from the following facilities: pools (*N* = 1091), hotels (*N* = 333), camping sites, (*N* = 331), ferries (*N* = 68), cooling towers (*N* = 59), apartments (*N* = 26), and rehabilitation centers (*N* = 24). Considering the share of positive samples per site, *Legionella* was most frequently found in cooling towers and rehabilitation centers (33.9% and 33.3%, respectively), in a similar percentage in camping sites, ferries, and apartments (between 19.2 and 21.8%), less frequently in hotels (11.7%), and the least frequent in pools (1.2%) (Figure 1).

Isolates belonging to *L. pneumophila* serogroups 2–14 prevailed in the studied samples (76.0%). Isolates of the most virulent *L. pneumophila* SG 1 were less frequent (22.2%), while only 1.8% of isolates were identified as *Legionella* spp. In total, in 38 of the 171 identified, the pathogen was *L. pneumophila* SG 1. Of the 38 identified strains of *L. pneumophila* SG 1, 16 of were found in hotels (42.1%), 9 in pools (23.7%), 5 in apartments (13.2%), 4 in camping sites (10.5%), and 2 in cooling towers (5.3%). Only one positive *L. pneumophila* SG 1 sample (2.6%) was found in ferries and rehabilitation centers (Figure 1). The highest percentage of *L. pneumophila* SG 1 isolates in *Legionella*-positive samples was found in apartments (5/5; 100%) and pools (9/13; 69.2%). On the other hand, SGs 2–14 were dominantly present in camping sites (68/72; 94.4%), ferries (13/14; 92.9%), cooling towers (18/20; 90.0%), and hotels (22/39; 56.4%).

**Figure 2:**
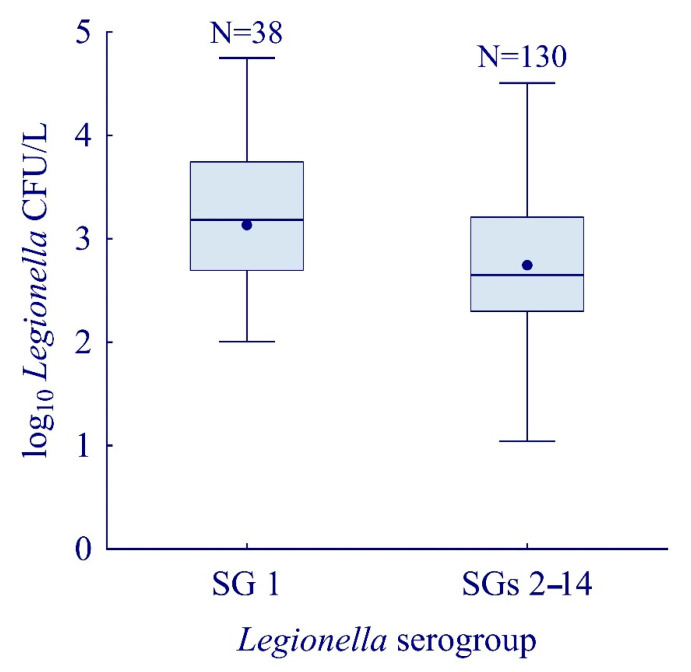
*Legionella* load in relation to strains. Boxplots show median (line), mean (point), quartiles (boxes), and non-outlier *L. pneumophila* range (whiskers).

Furthermore, there is a statistically significant difference between the concentrations in which specific serogroups appear in various water systems (M–W U, Z = −2.88; *p* = 0.003). Serogroup 1 of the *L. pneumophila*-positive water samples carried between 1.0 and 4.7 log CFU/L (median 3.2 log CFU/L), while in SGs 2–14-positive samples, concentration ranged between 0.8 and 4.9 log CFU/L (median 2.7 log CFU/L) (Figure 2).

### 3.2. Heterotrophic Plate Counts (HPCs) in Relation to the Presence of Legionella

The suitability of Heterotrophic Plate Count (HPC) as a warning sign of the presence of *L. pneumophila* was also assessed. This parameter is usually used as an indicator of general water quality as well as for the assessment of the efficiency of the disinfection treatment. Routine microbiological testing is determined using YEA as a nutrient media. YEA is a nutrient-rich medium that allows recovery of a wide range of bacteria. Besides YEA, sheep BA is also used for HPC determination. BA is a nutrient-rich medium that simulates the physiological human condition. It supports the growth of all clinically important bacteria that possess water-borne virulence factors [38,39].

**Figure 3:**
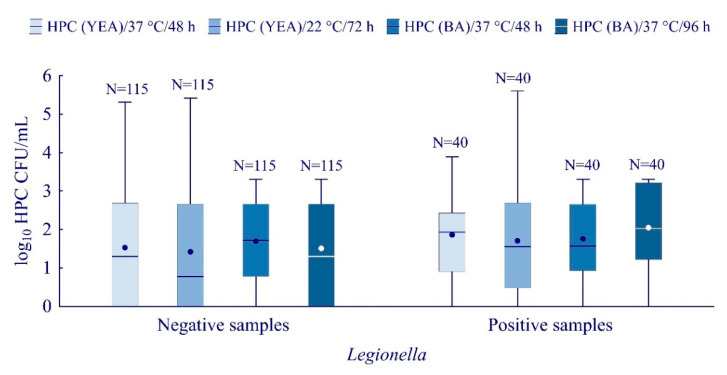
Heterotrophic Plate number (HPC) determined by using different agar and incubation conditions. Boxplots show median (line), mean (point), quartiles (boxes), non-outlier range (whiskers).

A statistically significant difference (M–W U, Z = −2.30, *p* = 0.02) in HPC values and the positive/negative *Legionella* samples were determined using sheep BA and incubation at 36 °C ± 2 °C for 96 ± 4 h (Figure 3). HPC have been positively associated with *Legionella* concentration only under the aforementioned incubation conditions (rs = 2.0, *p* < 0.05). For 44 h of BA incubation and using YEA as a nutrient media (regardless of incubation conditions), no significant correlation between HPC and concentration of *L. pneumophila* was observed.

### 3.3. Influence of Physical and Chemical Factors on Legionella Survival

**Figure 4:**
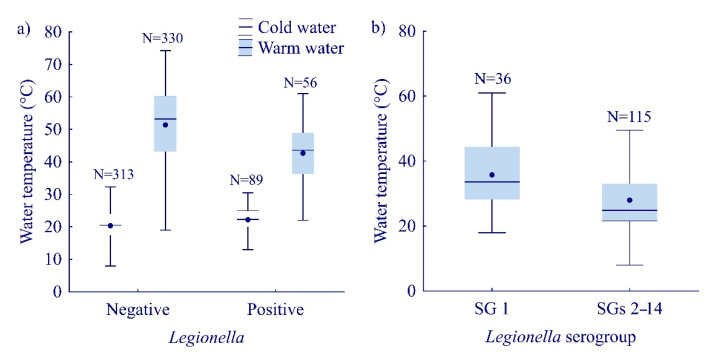
Water temperature in relation to: (**a**) *Legionella* presence; (**b**) *Legionella* strains (serogroups 1 vs. 2–14). Boxplots show median (line), mean (point), quartiles (boxes), non-outlier range (whiskers).

According to our results, *Legionella* is found in systems with cold water within a temperature range of 8.0 to 35.1 °C. At the same time, it was isolated from warm water systems ranging from 22.0 to 61.0 °C. An M–W U test confirmed a statistically significant difference in the median temperature among the *Legionella*-positive and -negative samples, for both cold and warm water environments (M–W U, Z = 3.30, *p* < 0.0001 and Z = −5.31, *p* < 0.0001, respectively). The median of cold-water temperature of the samples that tested positive for *Legionella* was 1.8 °C higher compared to the negative samples. Likewise, the median of warm water temperature of the *Legionella*-positive samples was 9.8 °C lower than the negative samples (Figure 4a).

Considering the prevalence of *L. pneumophila* serogroups in relation to temperature, it appears that, in samples with a confirmed strain of *L. pneumophila* serogroup 1, the median temperature values were 8.8 °C higher compared to the samples that tested positive to SGs 2–14 (M–W U, Z = −3.70, *p* < 0.0002) (Figure 4b).

**Figure 5:**
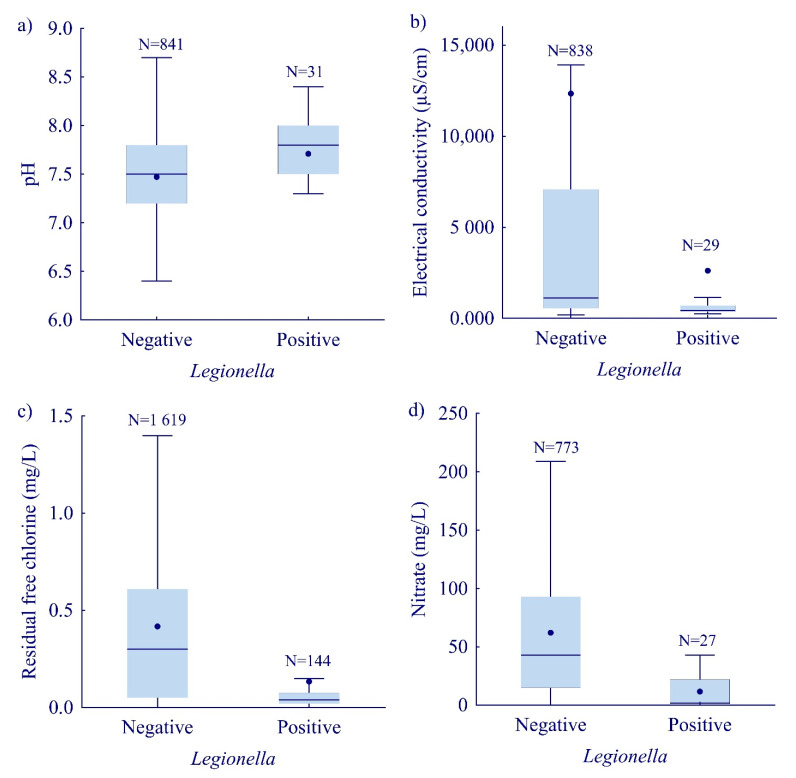
*Legionella* presence in relation to (**a**) pH value, (**b**) electrical conductivity, (**c**) concentration of residual free chlorine, (**d**) concentration of nitrate. Boxplots show median (line), mean (point), quartiles (boxes), non-outlier range (whiskers).

The median values of electrical conductivity (M–W U, Z = −4.36, *p* < 0.0001), nitrate concentration (M–W U, Z = −5.74, *p* < 0.0001), and the concentration of free residual chlorine (M–W U, Z = −10.00, *p* < 0.0001) were lower in samples positive for *Legionella* compared to the negative ones. This was found to be in contrast with the pH values (M–W U, Z = 3.42, *p* = 0.0006) (Figure 5a–d).

Our results show significantly higher pH values for *Legionella*-positive samples (M–W U, Z = 3.41, *p* = 0.0006), ranging from 5.2 to 8.4, though for negative samples the range was wider, from 3.1 to 9 (Figure 5a). Correlation analyses revealed a positive association between the *Legionella* count and pH values. The results of our study indicate that lower electrical conductivity supports the survival of *Legionella* (Figure 5b).

A lower median value of free-chlorine concentration was shown in *L. pneumophila*-positive samples (Figure 5c). In one-third (48/144) of the samples that tested positive for *Legionella*, the concentration of free residual chlorine was below or at the detection limit of the method (≤0.02 mg/L). The highest proportion of samples (63.2%, 91/144) measured a concentration of chlorine at lower levels (0.03–0.1 mg/L). Only 4.9% (7/144) of the samples showed a chlorine concentration of 0.2–0.5 mg/L, while 7.6% (11/144) of the samples measured a concentration that was above 0.5 mg/L. Hence, in two-thirds of the samples, *Legionella* was present simultaneously with chlorine. However, the correlation between free residual chlorine and the concentration of *Legionella* was significantly negative (rs = −0.24, *p* < 0.05).

The median values of the nitrate concentration were lower in the *L. pneumophila*-positive samples (Figure 5d).

### 3.4. Sequence-Based Typing (SBT) and Phenotypic Characterization

One of the aims of our study was to identify the presence of SBT types most commonly circulating in the Primorje-Gorski Kotar County. Nine bacterial isolates from different water samples were typed by SBT analysis. The allelic profile of the seven alleles (*flaA*, *pilE*, *asd*, *mip*, *mompS*, *proS*, and *neuA*) was obtained for each isolate. Six different allelic profiles were identified, and six ST groups were defined. Prevalent ST 1317 exhibited the profile “16, 21, 12, 19, 31, 21, 215” (Table 2). The allelic profiles obtained from our samples were already present in the EWGLI SBT database. The same nine isolates of *L. pneumophila* were used to determine phenotypic characterization. The isolates presented different phenotypic characteristics, but the most common phenotypes were Chicago 8 and Oxford/Olda (Table 2).

In our study, we also investigated whether a change would occur in the allelic profile and/or phenotype of the isolate after a pasteurization treatment at 53 °C for 10 min or after cultivation in *A. castellanii*. *Legionella* samples with an identified allelic profile underwent a pasteurization treatment as well as cultivation in amoebae, after which their phenotype was re-determined. To confirm the genetic changes, the isolates from the hotel (grown in amoeba or pasteurized) were selected for gene sequencing. Our results show that the pasteurization treatment and growth in amoeba do not affect the allelic profile and phenotype of the bacterium (Table 2).

### 3.5. The Growth of Legionella in Amoeba A. castellanii

Representative samples of *L. pneumophila* isolated from different water systems were used to determine growth kinetics in amoeba cells. *A. castellanii* cells were infected with *L. pneumophila* (MOI 100) and incubated at 27 °C for 10 days. At 0, 2, 4, 6, 8, and 10 days after infection, the number of intracellular bacteria was determined by the CFU method.

**Figure 6:**
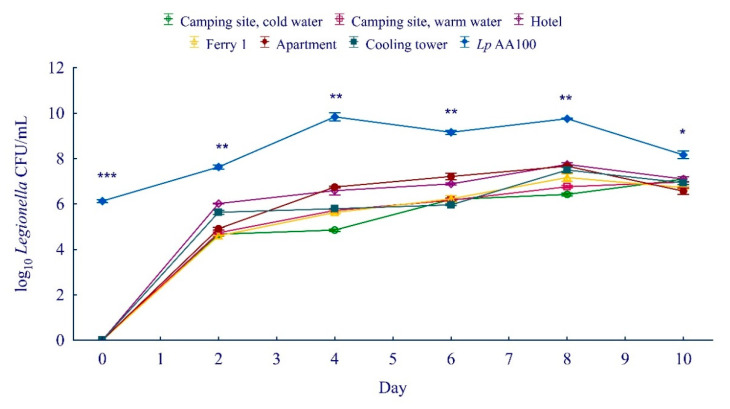
The growth kinetics of environmental *L. pneumophila* in *A. castellanii* during 10-day period. *L. pneumophila* AA100 was used as control. Student’s *t*-test, * *p* < 0.05, ** *p* < 0.01, *** *p* < 0.001 were accepted as significantly different from control sample.

Our results show that, at day 0 of the amoeba infection, environmental *L. pneumophila* did not replicate within *A. castellanii*. In contrast, *L. pneumophila* AA100 started proliferation and increased up to 10^6^ CFU/mL at day 0 of infection (Figure 6, Student’s *t*-test, *p* < 0.001). Environmental *L. pneumophila* induced replication in *A. castellanii* 2 days after infection and growth in amoeba increased up to day 8, after which the number of bacteria started decreasing. The number of intracellular *L. pneumophila* from the apartment was 8.3 × 10^4^ CFU/mL (2 days), 5.5 × 10^6^ CFU/mL (4 days), 2.0 × 10^7^ CFU/mL (6 days), 4.7 × 10^7^ CFU/mL (8 days), and 4.0 × 10^6^ CFU/mL (10 days) after infection (Figure 6). This was significantly different compared to the control samples (*L. pneumophila* AA100, Student’s *t*-test, *p* < 0.01 or *p* < 0.05) where the number of bacteria was higher during every time period: 4.4 × 10^7^ CFU/mL (2 days), 7.5 × 10^9^ CFU/mL (4 days), 1.5 × 10^9^ CFU/mL (6 days), 5.7 × 10^9^ CFU/mL (8 days), and 1.7 × 10^8^ CFU/mL (10 days). Environmental *L. pneumophila* showed reduced entry and replication in amoebae during a 10-day period, compared to control bacteria.

### 3.6. Pasteurization Treatment of Agar-Grown and A. castellanii-Grown Legionella

*L. pneumophila* displays an ability to survive in purpose-built water systems, despite routine disinfection treatment. In our study, we determined the highest temperature at which *Legionella* in the samples taken in Primorje-Gorski Kotar County can survive. Tap water samples were inoculated with six *Legionella* isolates in a concentration of 10^6^ CFU/mL for use in pasteurization trials. Pasteurization treatment at 63.5 and 55 °C for 10 min resulted in bacterial death. The highest temperature at which environmental *Legionella* isolates from this study can survive was 53 °C when treated for 10 min. All the bacteria survived heat treatment at 50 °C for 10 min, while only two out of the six tested *L. pneumophila* (autocamp cold and warm water) bacteria were killed after treatment at 50 °C for 30 min.

**Figure 7:**
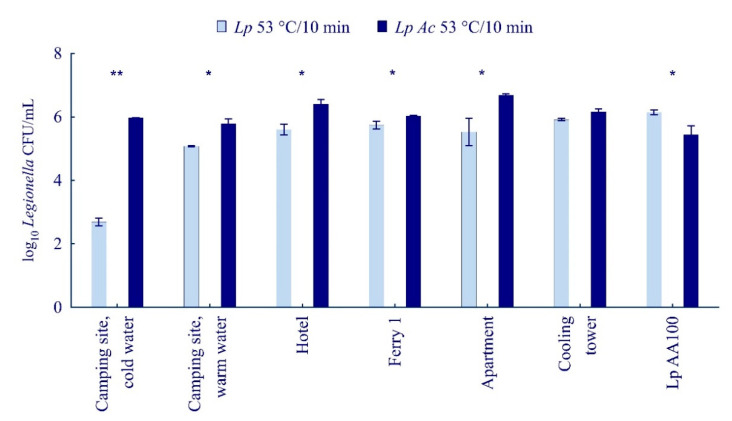
The number of *Legionella* CFU of agar-grown *L. pneumophila* (*Lp*) and *A. castellanii*-grown *L. pneumophila* (*Lp* AC) samples after pasteurization treatment at 53 °C for 10 min. *L. pneumophila* AA100 was used as the control organism. Student’s *t*-test, * *p* < 0.05, ** *p* < 0.01 were accepted as significantly different from control sample.

Furthermore, we investigated the number of agar-grown and amoeba-grown bacteria after heat treatment at 53 °C for 10 min; *L. pneumophila* AA100 was used as control. After heat treatment at 53 °C for 10 min, the number of agar-grown *L. pneumophila* was: 5.3 × 10^2^ CFU/mL for autocamp cold water, 1.2 × 10^5^ CFU/mL for autocamp warm water, 4.7 × 10^5^ CFU/mL for hotels, 5.9 × 10^5^ CFU/mL for ferries, 7.7 × 10^5^ CFU/mL for apartments, and 8.2 × 10^5^ CFU/mL for cooling towers (Figure 7). The number of amoeba-grown bacteria after pasteurization treatment was significantly higher (Student’s *t*-test, *p* < 0.01 or *p* < 0.05) compared to the number of agar-grown bacteria: autocamp cold water 9.2 × 10^5^ CFU/mL, autocamp warm water 6.8 × 10^5^ CFU/mL, hotel 2.8 × 10^6^ CFU/mL, ferry 1 1.1 × 10^6^ CFU/mL, apartment 4.9 × 10^6^ CFU/mL, and cooling tower 1.5 × 10^6^ CFU/mL. In contrast to the aforementioned results, *L. pneumophila* AA100 grown in amoeba were more susceptible to destruction by pasteurization treatment compared to agar-grown bacteria. The number AA100 amoeba-grown bacteria after pasteurization treatment was 4.3 × 10^5^ CFU/mL, while the number of agar-grown bacteria was 1.4 × 10^6^ CFU/mL (Figure 7, Student’s *t*-test, *p* < 0.05). We can conclude that only the bacteria from environmental sources, after adaptation to the aquatic environment, and *A. castellanii* become more resistant to destruction by high temperature.

### 3.7. Cell Morphology

**Figure 8:**
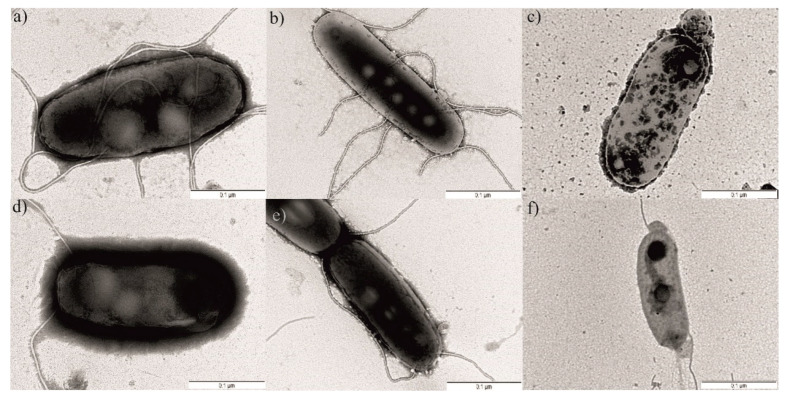
TEM images of *L. pneumophila* isolated from the ferry: untreated (**a**), grown in *A. castellanii* (**b**), and pasteurized at 53 °C for 10 min (**c**); *L. pneumophila* isolated from the hotel: untreated (**d**), grown in *A. castellanii* (**e**), pasteurized at 53 °C for 10 min (**f**).

The morphology of agar-grown, amoeba-grown, and pasteurized *L. pneumophila* was observed using TEM, by monitoring the changes in the structure of the cytoplasm, cell wall, and the existence of bacterial appendages such as flagella and pili (Figure 8).

*L. pneumophila* is a rod-shaped bacterium. Most of the bacteria had between one and three polar or lateral flagella. On the surface, *L. pneumophila* expressed pili of variable lengths. In addition, the cells showed the intact cell wall layer and compact and high electron-dense cytoplasm (Figure 8a,d). Interestingly, compared to the agar-grown cells, *A. castellanii*-grown *Legionella* cells did not show significant differences in cell morphology (Figure 8b,e).

However, the pasteurization treatment at 53 °C for 10 min caused changes in bacterial morphology (Figure 8c,f). The changes were manifested by cell wall damage and the separation of the cytoplasm from the cell wall in around 70% of the bacteria (Student’s *t*-test, *p* < 0.001). The morphology of the cytoplasm changed from high electron density to intermediate electron density. The disorganized cytoplasm also showed a tendency to clump in 67% of the pasteurized bacteria, when compared to the untreated bacteria (Student’s *t*-test, *p* < 0.001). Furthermore, the pili were lacking on the cell surface and the number of flagella was drastically reduced (Figure 8c,f), Student’s *t*-test, *p* < 0.01).

Examination of *L. pneumophila* by transmission electron microscopy demonstrated that growing in amoeba did not result in significant differences in cell morphology, while the pasteurization treatment showed significant changes compared to the untreated bacterial cells.

## 4. Discussion

During the study period (2013–2019), the average annual share of *Legionella*-positive samples in Primorje-Gorski Kotar County was 8.9% and was characterized by an upward trend. The largest percentage of *Legionella*-positive samples was found in cooling towers and rehabilitation centers (around 34%), while the smallest number in swimming-pool waters (1.2%). In a similar study conducted in Italy from 2014 to 2017 [40], *Legionella* was most frequently found in hotels (57.1%), followed by sport centers (41.2%), retirement homes (28.6%), and camping sites (12.5%). A low share of positive pool water samples was also revealed in an Italian study [41], in which *Legionella* spp. was found in 4.2% (2/48) of analyzed pool water samples. Therefore, it was concluded that the risk of being infected in such places is higher when showers are used, rather than by recreation in swimming pools.

In this study, *L. pneumophila* SGs 2–14 was the most commonly identified strain (76%) isolated. That is generally consistent with the results of numerous other studies, in which serogroups 2–14 accounted for over 50% of isolates [42]. The highest proportion of serogroup 1 was found in apartments (100%) and swimming-pool waters (69.2%). *L. pneumophila* was found in 13 of the 1091 examined pool water samples, of which 12 of them were of whirlpool spa pool type, where higher water temperature and aerosol formation in hydro-massage systems enhance the risk of infection [43]. According to the national regulations for swimming-pool water quality, in swimming pools with a water temperature ≥23 °C and the possibility of aerosolization of water, the concentration of free residual chlorine must be between 0.7 and 1.0 mg/L. Of the 12 *L. pneumophila*-positive spa water samples, all had a temperature greater than 30 °C, while only three samples (25%) had a free residual chlorine concentration greater than the required minimum of 0.7 mg/L. These results indicate the need for increased disinfection of the spa while meeting the prescribed criteria. Considering the prevalence of *L. pneumophila* strains, based on the type of facilities, an unequal distribution of serogroups can be perceived. Our results are consistent with those of a French study (2001–2002), which found a proportion of *L. pneumophila* SG 1 in samples from environmental sources of 28.2% [44]. The presence of *L. pneumophila* SG 3 (10.8%) was significant in the environmental samples, while non-*L. pneumophila* species, *L. anisa*, accounted for 13.8% of the isolates. In a study conducted in South Korea in 2010 [45], a different ratio of serogroups among *L. pneumophila* species was found in environmental samples. It showed a higher share of *L. pneumophila* SG 1 (54.7%) when compared to *L. pneumophila* SGs 2–15 [45]. On the other hand, research carried out by Sikora, et al. [46] in 26 Polish facilities (hospitals and public buildings) during the 2007–2010 period revealed only the prevalence of *L. pneumophila* SGs 2–14.

The *Legionella* load in *L. pneumophila* SG 1-positive samples was higher compared to the *L. pneumophila* SGs 2–14-positive samples. These results are in contrast with those of a study conducted in Rome, Italy (2014–2017), in which the *Legionella* load was significantly higher in samples testing positive for *L. pneumophila* SGs 2–14 [40]. In the Italian study, *Legionella*-positive samples were mostly collected from larger buildings (hotels and sports centers) supplied with groundwater via the public distribution system, whereas in our study samples were collected from smaller distribution systems of camping sites using surface water. Considering the known higher virulence of serogroup 1, this could be taken into account when assessing the risk of *Legionella* contamination in camping sites [47]. In 35.5% (N = 61) of the positive samples, the acceptable value of 1000 CFU/L for *L. pneumophila* was exceeded (according to the proposal of the New Water Consumption Directive for water from domestic distribution). That share included more than half (20/38; 52.6%) of *L. pneumophila* SG 1 strain and less than a third (39/130; 30.0%) of the SG 2–14 isolates.

In our study, the *Legionella* load was found to be positively associated with the HPCs values determined by the pour plate method using sheep BA at 36 °C for 96 h. Overall, the correlation between HPCs values and the *Legionella* load is inconsistent throughout the literature. Thus, this relationship requires further research [21,40,48].

*L. pneumophila* multiply in the range of temperatures between 20 and 48 °C [49,50]. The highest temperature at which *Legionella* from Primorje-Gorski Kotar County can survive was 53 °C when treated for 10 min. This is consistent with the results of another study conducted in Southern Croatia, which found that water temperature higher than 54 °C is protective, while the temperature range of 44–54 °C positively affects the colonization of bacteria [24]. Our results showed that *Legionella*-positive samples were frequently collected from cold water distribution systems with higher water temperature and from warm water distribution systems with lower water temperature. The results of our study are in accordance with the results of a study conducted in Greece by Dimitriadi and Velonakis [51]. In their study, they measured the mean temperature of hot water of the *Legionella*-positive samples and found that it was 4.4 °C lower compared to that of the negative samples. A study conducted in Split-Dalmatian County by Rakić and Štambuk-Giljanović [52] included measurement of the lower median value of temperature in *Legionella* spp.-positive samples (by 7.0 °C) compared to the negative samples (47 °C vs. 54 °C). In contrast, in research carried out in Iran, Tabatabaei, et al. [53] found that the average temperature of *Legionella* spp.-positive water samples was 6.6 °C higher compared to the average temperature of the negative samples (44.2 °C vs. 37.6 °C). In other words, both medians fell within the temperature range that is favorable for bacterial growth. In samples with a confirmed strain of *L. pneumophila* SG 1, the median temperature values were 8.8 °C higher compared to the samples that tested positive for SGs 2–14. A higher incidence of *L. pneumophila* SG 1 in warm water was also found by a Korean study conducted by Lee et al. [45]: SGs 2–14 that occurred to a greater extent in cold water (61.8%), and SG 1 in warm water (58.8%).

In our study, the survival of *Legionella* was enhanced in the samples with higher pH values, while nitrate concentration, electrical conductivity, and free residual chlorine significantly reduced *Legionella* survival. In general, it is believed that higher pH values (6.0–8.0) enhance the survival rate of bacteria [52]. The survival of *Legionella* at lower electrical conductivity was also found by a study carried out by Lasheras, et al. [54], although in contrast with the results of Karki and Cann [55].

It is known that the correlation between free residual chlorine and *Legionella* concentration is significantly negative. In our study, however, *Legionella* was present simultaneously with chlorine in two-thirds of the samples. This indicates the possibility of bacteria being protected within the biofilm, thus reducing the effect of chlorine disinfection [52]. Rakić et al. [24,52], in their work, carried out in the Split-Dalmatia County (Croatia), did not determine the correlation between chlorine concentration and *Legionella* presence and suggested possible resistance of *Legionella* spp. to chlorine.

Most of the environmental samples in our study belonged to ST 1317, while the most common phenotypes were Chicago 8 and Oxford/Olda. In a study conducted in Northern Sicily, SBT analysis of 86 *L. pneumophila* SG 1 isolates, from water distribution systems, showed that most samples belonged to ST1 [56]. The ST1, which we have found in a wellness center, was also the most frequently detected ST among clinical isolates in Germany [57], and is considered as the most common SBT in Europe. In a study conducted in China, *L. pneumophila* SG 1 isolates from cooling towers, hot springs, and drinking-water systems were analyzed by SBT. The results of the study also show that ST1 was the most prevalent ST in China, accounting for 49.4% of the analyzed strains [58].

In 2010, there was an outbreak of legionellosis in a Slovenian nursing home. The SBT analysis showed an identical sequence type 23 in the water supply system and the isolates from the patients [59]. In a study in Catalonia, 528 *L. pneumophila* isolates from the environment (cooling towers, hotels, hospitals, water distribution system, spas, sprinklers, tanks, pools) and the clinic were described and compared using SBT and monoclonal antibody subgrouping, and 32% of the clinical isolates belonged to ST1, ST23, and ST37, while 40% of the environmental isolates belonged to ST1 and ST284 [60]. The most abundant subgroups of clinical isolates were Philadelphia (26.61%), Knoxville (19.27%), and OLDA (14.68%), while OLDA (33.1%) were the most frequent environmental isolates [60]. A recent study in the Croatian northern Adriatic presented a case of LD in a patient who visited a spa complex in a hotel, where the isolate from the water belonged to ST82 and the Allentown/France MAb subgroup [61].

*L. pneumophila*, as well as numerous other bacteria, have shown the ability to enter and multiply in *A. castellanii* cells [16]. A recent study also showed that amoebae likely promote survival of some bacteria in aquatic environments [62]. After the life cycle in an amoeba, *L. pneumophila* becomes more resistant to destruction by pasteurization treatment compared to agar-grown bacteria. Our results show that pasteurization treatment and growth in amoeba do not affect the allelic profile and phenotype of the bacterium, while examination by transmission electron microscopy demonstrated significant changes in the morphology of pasteurized bacterial cells. *A. castellanii*-grown *Legionella* cells did not show significant differences in cell morphology compared to agar-grown cells. This is in contrast with a recent study on *Francisella novicida*, in which the morphology of *F. novicida* changed after growth in *A. castellanii*; this was manifested through cell wall damage, the reduction in cytoplasm electron density, and leakage of intracellular material [32]. However, our results are consistent with the *Legionella* counts, which showed that amoeba-grown *Legionella* becomes more resistant to pasteurization treatment.

## 5. Conclusions

*Legionellae* are opportunistic pathogens that are widely distributed in aquatic environments. They survive in aqueous media at varying temperatures, electrical conductivity, pH, and nutrient levels, as well as varying concentrations of free residual chlorine in treated waters. The risk of *Legionella* proliferation is increased in cold water distribution systems with a temperature above 22.3 °C and warm water systems with a temperature below 43.5 °C. The risk of *Legionella* proliferation is also increased in water with electrical conductivity less than 419 µS/L, nitrate concentration less than 1.7 mg/L, free residual chlorine concentration less than 0.04 mg/L, and pH greater than 7.8. The sources of *Legionella* transmission to humans are well characterized, and all of the sources examined in this study involve aerosolization of water contaminated with *Legionella*. Drinking water is considered the most important source of *Legionella* transmission, especially in complex systems such as camps, where water supply pipes are often upgraded and not laid deep enough in the ground, making it difficult to maintain the required temperatures. In such systems, the risk is additionally increased due to the higher *Legionella* load of serogroup 1. It is important to monitor serotypes and genotypes from clinical samples in order to link them with the source of infection in environment, especially in tourist-attractive countries. Our results showed that most of the samples from the apartments belong to a highly infectious serogroup 1, indicating the importance of preventive measurements to control the infection. Of all *Legionella*-positive drinking-water samples, 35.5% were above the acceptable new EU Water Consumption Directive level of 1000 CFU/L for *L. pneumophila. Legionellae* survive and thrive in vesicles after being ingested by amoebae in water resources. The data from this study contribute to the understanding of the effects of increased resistance of amoeba-cultured *L. pneumophila* from amoeba cultures to pasteurization treatment.

The fact that amoeba-grown *L. pneumophila* becomes more resistant to pasteurization treatment suggests that growth in protozoan cells may be responsible for the development of the disease in humans, and future studies should focus on a better understanding of the way in which bacterial growth influences the pathogenesis of the disease. The results of this study will be useful for managing *Legionella* in water systems of different facilities.

## Figures and Tables

**Table 1:** Number of total samples analyzed, number and share of *L. pneumophila*-positive samples.

Year	*N*	*N* *Legionella* Positive	% *Legionella* Positive
2013	333	0	0
2014	93	0	0
2015	96	0	0
2016	185	17	9.2
2017	302	42	13.9
2018	412	60	14.6
2019	511	52	10.2
Total	1932	171	

**Table 2:** Serogroups, STs, allelic profiles, and phenotypes of the *L. pneumophila* environmental samples isolated in the Primorje-Gorski Kotar County (Croatia).

Sample	Type of Water Source/Disinfectant	*L. pneumophila*	*Lp* SG	ST	Allelic Profile	Phenotype
Hotel	groundwater/ClO_2_	isolate from water	1	82	5,1,22,10,6,10,6	Allentown
Apartment	surface water	isolate from water	1	62	8,10,3,15,18,1,6	Knoxville
Camping site, cold water	surface water/ClO_2_ + NaClO	isolate from water	7	1317	16,21,12,19,31,21,215	Chicago 8
Camping site, warm water	surface water/ClO_2_ + NaClO	isolate from water	7	1317	16,21,12,19,31,21,215	Chicago 8
Ferry 1	groundwater/Cl_2_	isolate from water	7	1317	16,21,12,19,31,21,215	Chicago 8
Cooling tower	groundwater/ClO_2_	isolate from water	10	1365	1,4,3,28,1,1207	Leiden 1
Ferry 2	groundwater/NaOCl	isolate from water	1	986	1,14,16,16,15,13,2	Oxford/Olda
Wellness	groundwater/Cl_2_	isolate from water	1	1	1,4,3,1,1,1,1	Oxford/Olda
Rehabilitation Centre	groundwater/ClO_2_	isolate from water	1	62	8,10,3,15,18,1,6	Oxford/Olda
Hotel, 53 °C/10 min	groundwater/ClO_2_	isolate from water- heat treated	1	82	5,1,22,10,6,10,6	Allentown/France
Hotel—*Lp* grown in amoeba	groundwater/ClO_2_	isolate from water- grown in amoeba	1	82	5,1,22,10,6,10,6	Allentown/France

## Data Availability

The data presented in this study are available on request from the corresponding author.
